# Orthographic character complexity modulates dynamic neural activity in skilled handwriting

**DOI:** 10.1111/bjop.12742

**Published:** 2024-10-05

**Authors:** Leisi Pei, Werner Sommer, Guang Ouyang

**Affiliations:** ^1^ Department of Curriculum and Instruction, Faculty of Education and Human Development The Education University of Hong Kong Hong Kong SAR China; ^2^ Department of Psychology Humboldt‐Universität Zu Berlin Berlin Germany; ^3^ Department of Physics Hong Kong Baptist University Hong Kong SAR China; ^4^ Faculty of Education National University of Malaysia Kuala Lumpur Malaysia; ^5^ Complex Neural Signals Decoding Lab, Faculty of Education The University of Hong Kong Hong Kong SAR China

**Keywords:** Chinese handwriting, EEG, ERP, motor programming, naturalistic paradigm

## Abstract

Handwriting is an outstanding case of a highly complex and efficient fine motor skill. However, little is known about its neural underpinnings during continuous handwriting production. In the present study, we examined the effects of orthographic character complexity (i.e. the stroke number of a Chinese character) on both neural and behavioural activities during an EEG‐based naturalistic fluent sentence‐handwriting task from 102 adult Chinese native speakers. For each written character, the interval between finishing the preceding character and its onset (inter‐character interval) as well as the amplitudes of the onset‐synchronized event‐related potential (ERP) in pre‐ and post‐onset time windows was defined as dependent variables. The effects of character complexity and other confounding factors were analysed with linear mixed models. Character complexity increased the inter‐character interval and significantly affected ERP amplitudes in both pre‐ and post‐onset time windows. The ERP pattern in the pre‐event time window exhibited a dipole‐like activation in the left motor cortex, and its amplitude increased with character complexity in line with the documented relationship between the lateralized readiness potential and motor complexity. This study demonstrates the feasibility of studying neurocognitive processes in complex naturalistic motor tasks and extends our knowledge about the dynamic pattern of handwriting‐related neural activities.

## BACKGROUND

In modern societies, handwriting is an essential but highly complex manual motor skill (Palmis et al., 2017). For an average person, fluid handwriting requires years of extensive practice, resulting in a high level of automaticity (McHale & Cermak, 1992), usually leading to subjective unawareness of the complexity of the underlying processes. From a motor control system perspective (Teulings, 1996), there are at least three characteristics that reflect the functional complexity of handwriting. The first characteristic is the highly coordinated sensory‐motor system supporting the fine motor execution of producing delicately organized movement patterns. The second characteristic is the complexity of written patterns that need to be retrieved instant by instant from long‐term memory. These patterns include the visual form and the motor programming/preparation of the complex sequential movements (Hulstijn & van Galen, 1983, 1988). The demands on these characteristics are more pronounced in scripts with more complex visual forms. For example, as compared to letters in alphabetic writing systems, the logographic Chinese characters are composed of a number of interlaced strokes that have to be written within imaginary two‐dimensional squares of identical size. The complexity of Chinese character is thus primarily determined by the total number of strokes composing the character (Liversedge et al., 2014; Ma & Li, 2015; Wang et al., 2014). Third, skilled handwriting can be performed in a seamless, highly fluent, online manner.

Given the complexity of the handwriting skill and its significance for human life, it is important to study the above‐mentioned characteristics in detail in order to understand their fundamental processes, development, and disorders. Previous research has outlined the sequence of functional processes involved in handwriting (Graham & Weintraub, 1996; Palmis et al., 2017). Briefly, the orthographic information about a character/word to be written is first retrieved from long‐term memory according to linguistic cues (e.g., semantic or phonetic information). A specific motor sequence associated with the target character/word, encapsulated as an abstract motor programme, is then retrieved from long‐term motor memory. Next, concrete parameters specifying the sequence, size and speed of the strokes are encapsulated and transmitted to the motor programme for execution (Plamondon et al., 1990; Plamondon & Maarse, 1989; van Galen & Teulings, 1983). Based on such processes, it is assumed that the complexity of the content to be written (e.g., a character) is a major factor shaping motor behaviour as complexity may affect both the retrieval and the execution of the motor programme. The complexity of handwriting content has been shown to affect the interval between content presentation and motor execution (Henry & Rogers, 1960; Jahanshahi, 2003), supporting its influence on retrieval from memory and motor preparation. Studying the effects of character complexity may help to delineate the basic unit of motor programming in handwriting. For instance, the movements of writing a Chinese character can be decomposed into different hierarchical levels (Sternberg et al., 1990), including single strokes, radicals (i.e. a group of strokes, which serve as a phonetic or semantic indicator of a character), parts and the characters as a whole (Chen & Cherng, 2013; Sternberg et al., 1990). If the complexity of individual Chinese characters – usually operationalized as the total number of strokes – exerts significant effects on behavioural measures or their neural correlates, it may indicate that characters are at least one level at which units of motor programmes are formed.

Although there has been extensive research into the effects of motor action complexity on motor programming (Jahanshahi, 2003; Zelaznik & Hahn, 1985), most studies have been carried out in settings where isolated motor responses were required to obtain response times (Klapp et al., 1979; Sternberg et al., 1978). In such paradigms, the effect of the complexity of motor programming is investigated based on reaction times (RTs; see Jahanshahi, 2003, for a review). To accommodate RT paradigms, most motor tasks are relatively simple with short sequences so that participants can quickly learn after several repetitions (Doyon et al., 2018). However, the discrete nature of such motor responses may fundamentally differ from complex naturalistic but automatized movements as required in handwriting. Apart from being simple and discrete, the motor sequences ‘temporally’ learned in lab settings may engage different neural processes from the naturalistic motor sequences that have already been highly practiced and consolidated in long‐term memory (Wymbs & Grafton, 2014). Therefore, it is necessary to study the complexity effect on motor programming of highly practiced, naturalistic, self‐paced motor tasks that retain the characteristics of online processing in a continuous manner.

Although handwriting can serve as an ideal venue to study motor programming under naturalistic activity, most studies relating handwriting to brain activity have either (1) adopted non‐naturalistic discrete paradigms (De Kleine & Van der Lubbe, 2011; Li et al., 2021; Richards et al., 2009; Swett et al., 2010) or (2) studied the gross neuroanatomical associations of naturalistic handwriting activity without going into details (Askvik et al., 2020; Katanoda et al., 2001; Longcamp et al., 2014; Yang et al., 2019). Previous neuroanatomical research has associated an extensive, mainly left hemispheric, cortical and sub‐cortical network with handwriting, including both writing‐specific and non‐specific motor and linguistic processes (Planton et al., 2013). These neuroanatomical correlates of handwriting provide an invaluable background for studies concerning the neural systems underlying handwriting. However, the temporal resolution (at the level of seconds) from the neural anatomical research is not sufficient for studying the fast dynamics of handwriting, which may require neural electrophysiological recording. To the best of our knowledge, with the exception of a pioneer work (Pei et al., 2021), there is no study that employs electrophysiological signal technology (EEG) to investigate elementary dynamic motor action in naturalistic handwriting. In the previous study (Pei et al., 2021), the researchers successfully extracted a well‐structured event‐related potential (ERP) component associated with the initial point of writing a stroke during naturalistic handwriting process based on a self‐developed EEG‐tablet synchronization system. This neural component captures the time course of neural activity associated with elemental process of handwriting production, providing a foundation to investigate the potential effects of various cognitive factors (e.g., script complexity) on the temporal neural dynamics underlying handwriting production. This methodology will be employed also in the current study.

Given the fast pace of fine‐grained motor activity in handwriting, EEG appears to be particularly suitable for studying the elementary neural activities and dynamics underlying or correlated with this mental and motor activity (Pei et al., 2021). EEG research has disclosed a number of ERP components associated with simple motor processes from discrete motor tasks (see Shibasaki & Hallett, 2006, for a review). While the relations between these components and motor response complexity are largely unknown, the situation is better concerning the Bereitschaftspotential (BP) or readiness potential (RP). BP is a negative‐going potential at central scalp regions (Cui et al., 2000; De Kleine & Van der Lubbe, 2011). Its amplitude has been associated with the complexity of motor preparation (e.g., Leuthold & Schröter, 2011). Worth mentioning is the lateralized readiness potential (LRP), the response hand‐related asymmetry of the BP. The LRP amplitude has also been related to response complexity (Hackley & Miller, 1995; Stief et al., 1998; Xu et al., 2015).

The overwhelming majority of previous research on BP is based on tasks that require discrete motor actions often in response to cue signals at long interaction intervals (Cui et al., 2000; Kitamura et al., 1993; Leuthold & Schröter, 2011; Mussini et al., 2021). These tasks strongly differ from highly practiced, naturalistic fine motor control activities as in handwriting, typing or playing a music instrument, where actions are highly fast‐paced and the neural activities associated with the sequential single action units are thus densely packed and strongly overlapping with each other on the time axis. Such highly compact motor actions leave little time for preparatory activity as reflected in the BP that usually unfold over 500 ms or more (Schurger et al., 2021). Therefore, it is necessary to know whether neural activity resembling preparatory motor activity (e.g., BP) can be identified in a compact naturalistic task, and if so, what are their characteristics.

As mentioned above, previous EEG research has identified close associations between BP/LRP and motor response complexity (Cui et al., 2000; Kitamura et al., 1993; Leuthold & Schröter, 2011; Mussini et al., 2021). Specifically, most studies have found an increased amplitude of BP or LRP with response complexity, which was seen to be due to more motor parameters needed to be programmed at the stage of motor planning or implemented before movement execution (Hackley & Miller, 1995; Leuthold & Schröter, 2011; Schmitz et al., 2019; Smulders et al., 1995; Stief et al., 1998; Xu et al., 2015). However, we argue that these effects cannot be directly extrapolated to a naturalistic task in which a great number of cognitive processes and motor activities are intertwined for several reasons: (1) It is unclear how much in advance motor processes are planned in highly compacted naturalistic tasks. (The naturalistic tasks in this article refer to tasks with the actual realization of all processes that would exist in a genuine naturalistic activity.) Therefore, examining the timing of relevant neural effects is informative for us to understand naturalistic scenarios. (2) The highly compacted activities in naturalistic tasks (e.g., online handwriting) may lead to interference and interactions between parallel subprocesses or overlapping adjacent processes, which are easier to separate in simple, discrete tasks. We therefore need to ask if the relevant effects such as those of complexity on BP/LRP/prevail in such contexts. (3) A highly skilled motor performance conducted in an online manner may recruit distinct processes – such as efficient online long‐term memory retrieval (Wymbs & Grafton, 2014) – that might be different from discrete tasks. Based on these rationales, it is of theoretical importance to assess complexity effects on neural and behavioural activities in naturalistic tasks, such as handwriting.

In a continuous task with compact motor actions like naturalistic handwriting, the complexity of motor actions varies in real time in a way that is associated with the complexity of written content. This is particularly the case in Chinese handwriting. Therefore, the specific relationship between BP and motor complexity can be used as additional evidence to assess the BP component in online, naturalistic handwriting. Taken together, we hypothesized that (1) the BP should be identifiable in a continuous naturalistic motor task after controlling for confounding factors in the compact actions and (2) BP increases with the complexity of motor actions in real time. To investigate the neurocognitive correlates of the online coordination of handwriting movements in a naturalistic mode and test these two hypotheses, we recorded synchronized EEG signals and kinematic handwriting streams from 102 adult participants. More specifically, we examined the effect of the complexity of Chinese characters on both the movement kinematics and neural activities associated with elementary motor processes. In the task, participants repeatedly wrote the same Chinese sentence in a natural way with a stylus on a tablet computer. The sentence was composed of eight Chinese characters with different complexities (quantified by the number of strokes composing each character). We recorded ERP time‐locked to the start of writing each character and calculated the inter‐character intervals (ICIs), defined as the time from finishing the last stroke of the preceding character to the onset of the first stroke of the character in question. ERPs and ICIs served as dependent variables. We applied linear mixed modelling to examine the effects of character complexity after regressing out major confounding factors arising from the naturalistic task. Based on previous research as reviewed above, we expected that character complexity increases ICIs and affects ERPs in both pre‐ and post‐character time windows due to their potential roles in affecting memory retrieval, motor preparation and execution. In particular, we expected the presence of a BP‐like ERP component in this naturalistic compact motor activity and its modulation by character complexity.

## METHODS

### Participants

The data used in this study were from a previous research project on handwriting and its relationships with other cognitive abilities. In the project, a total of 102 healthy adults recruited in Hong Kong participated in the experiment after giving written informed consents. The project was approved by the Human Research Ethics Committee (HREC) of The University of Hong Kong. All participants recruited in the project were native Chinese speakers who had received K‐12 education in mainland China and attained university education or above. Their handwriting experiences were highly dominated by simplified Chinese, as taught in mainland China. All participants were right‐handed and had little handwriting experience with their left hands. They reported normal or corrected‐to‐normal vision and no history of mental disease. None of the participants showed detectable motor impairments and all of them performed handwriting with smooth movements. During the experiment, the participants were required to perform a series of cognitive tasks, one of which was a handwriting task. The total task duration during EEG session was around 1 hr. In this study, we only used the data from the handwriting task. One participant was excluded due to adding extra characters in sentence writing, leaving 101 participants (mean age = 25.3 ± 3.8 years; 50 males).

### Apparatus and data recordings

**FIGURE 1:**
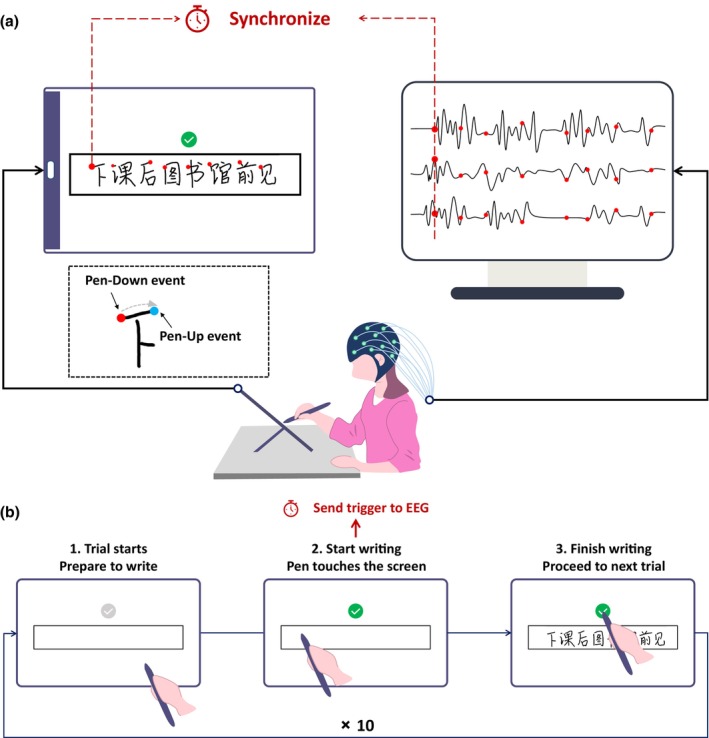
Experimental setup and task. (a) The co‐registration system that synchronizes the handwriting trajectories and EEG signals. Red dots denote the first pen‐down events of writing each character. Insert: Zoom‐in to the first character. The red and blue dots signify onset and offset of the first stroke, respectively. (b) One trial (of ten) where the designated sentence was written in a single horizontal line within the marked writing area of the tablet; the termination of each sentence was marked by tapping the check area.

The experimental setup is illustrated in Figure 1a. Participants wore an elastic EEG cap (actiCAP, Brain Products, Germany) in which 32 electrodes were placed in accordance with the 10–20 international system (Pivik et al., 1993). Electrodes were connected to an EEG amplifier system (BrainAmp, Brain Products, Germany) using the FPz electrode placed on the forehead (midway between the Fp1 and Fp2 electrodes) as initial common reference.

A tablet computer (HUAWEI MatePad Pro; screen resolution: 2560 × 1600 pixels; diagonal size: 10.8 inches; aspect ratio: 16:10) was placed in landscape orientation into a tablet holder, set to a slope of 40°, on the desk in front of the participant. An active stylus with 4096 levels of pressure sensitivity recorded the handwriting movements.

During the task, the handwriting trajectories on the tablet and EEG signals were simultaneously recorded at sampling rates of 60 Hz and 1000 Hz, respectively. The handwriting task was conducted through a self‐developed Android app. This app generates event logs for each point in the trajectory, including x/y coordinates, timestamp, force and state codes for the following events: pen‐down (contacting the tablet screen), pen‐move (moving across the screen) and pen‐up (separating from the screen). The handwriting and EEG data streams were synchronized through a self‐developed system that generates the time marker of the first pen‐down event of writing each sentence and sends it to the online EEG stream for synchronization. The entire technical setup adhered to the one used by Pei et al. (2021).

### Chinese handwriting task

In the Chinese handwriting task (see Figure 1b), participants first memorized a designated sentence and then wrote the sentence in a single horizontal line within a specified area on the tablet with their right (i.e. dominant) hands. This was repeated 10 times: After finishing writing a sentence, participants clicked the ‘submit’ button above the writing area and proceeded to writing the next sentence repetition. Each sentence repetition constitutes a trial. The designated Chinese sentence was ‘下课后图书馆前见’ (English translation: ‘See you after class in front of the library’). The eight characters of this sentence are very common and are to be mastered before Grade 4 according to the national curriculum standard of Mainland China.

It is noted that the handwriting task was the final task and the only task that required to produce highly complex movements in the EEG experiment. Before that, the participants completed five tasks that required them to make judgements by pressing left or right Ctrl key or draw lines on the tablet. To alleviate the potential impact of fatigue on task performance, the participants were instructed to take rests between tasks until they felt fit to continue. The entire EEG session lasted for about 1 hr.

To ensure homogeneity and high quality of handwriting production, participants were instructed to (1) write each character stroke‐by‐stroke and avoid scribbling, (2) keep stroke order consistent when writing the same character across trials, (3) keep character size consistent within and across trials, (4) not to correct writing errors but repeat the character and (5) to stop writing when running out of space in the designated writing area. To control the impact of muscle artefacts on the EEG recordings, the participants were required to avoid large body movements during task performance, including moving heads and body, stretching arms, hands or legs, or moving the handwriting arm away from the desk. These task instructions adhered to the ones used in the work of Pei et al. (2021).

### Data analysis

#### EEG preprocessing

EEG signals were preprocessed and analysed using MATLAB (Math Works, R2021a) and its EEGLAB plugin (Delorme & Makeig, 2004) by down‐sampling to 250 Hz and bandpass filtering with the inbuilt FIR bandpass filter between 1 and 45 Hz (zero‐phase, non‐causal, filter order: 827 data points, corresponding to 3.3 s). Bad electrodes, defined by variance greater than four times the median absolute deviations (MAD) across all electrodes, were interpolated. The mean and standard deviation of interpolated electrodes was 1.53 ± 0.99. No interpolation was performed on Fz and Pz electrodes (i.e. two electrodes used for statistical analysis). The EEG was re‐calculated to the common average reference, which has the advantage of being independent of any real electrode. Artefacts were removed by Independent Component Analysis and the MARA toolbox (Winkler et al., 2011). The probability threshold for automatic artefact removal in MARA was set at 0.5.

#### Labelling stroke information in Chinese characters

The sentence repeatedly written by the participants consisted of 8 characters, each composed of 3 to 11 strokes. The start and end points of each stroke were marked by the pen‐down and pen‐up events, respectively. In order to quantify this information, we developed a MATLAB app, labelling each stroke in terms of (1) its order within the character, (2) the ordinal number of the character within the trial (1–8) and (3) the ordinal number of the trial (1–10) to which the stroke belonged. Stroke order in a given character was identified individually because it slightly varied between participants. Trials containing writing errors, for example, additional characters or characters replacing incorrect ones, were excluded from analysis. In sum, each of the 101 included participants submitted 10 trials of sentence writing. Seven erroneous trials from seven different participants were identified and eliminated before the statistical analysis. Across all participants, the eliminated trials accounted for approximately 0.7% of the total number of trials. The app‐based stroke labelling provides rich information about handwriting kinematics and allows for studying the effects of many factors on behavioural or neural outcome.

#### Quantifying basic kinematic characteristics of handwriting

We quantified six representative kinematic features of the handwritten characters:

1. Actual stroke number: The average number of strokes in actual writing. In this study, a stroke was defined as the trajectory between a pair of adjacent pen‐down and pen‐up events as recorded by the tablet computer (Xiaolin & Dit‐Yan, 1997).
2. Length of a character: The total physical length of all strokes within a character. Stroke length was calculated as the Euclidean distances between every pair of adjacent writing points within the same stroke.
3. Duration of a character: The sum of durations for drawing the strokes within a character. Stroke duration was calculated as the time difference between the timepoints of the pen‐down and pen‐up events of writing a given stroke.
4. Max force of the first stroke: The maximal pressure of touch when writing the first stroke of the character. Each point on a handwriting trajectory is associated with a pressure value recorded by the tablet computer. Thus, the max force of the first stroke was calculated by finding the maximal pressure value recorded during the trajectory of writing the first stroke.
5. Mean velocity of the first stroke: The average velocity of writing the first stroke of a character, calculated by dividing stroke length by stroke duration (see definitions 2 and 3 above).
6. Mean velocity during character writing: The average velocity of writing all the strokes within a character, calculated by diving the length of a character by the duration of writing that character.

The six kinematic features were quantified for each character, trial and participant. First, for each participant, the means of the six features for each character were obtained by averaging across all trials after removing outliers. Then, means and corresponding standard deviations for each character were obtained by averaging across all participants after removing outliers. Finally, the means and corresponding standard deviations for each complexity group (see definition of complexity group in the following section) were calculated by averaging across the included characters. Data points that were more than 2* interquartile range (IQR) above the 3rd quartile or below the 1st quartile were defined as outliers and excluded before performing averaging and conducting statistical analysis. The six kinematic features were mainly used to provide more information to support the validity of grouping the characters into the two complexity groups.

#### Neural activation patterns synchronized with character writing

The first pen‐down events in each character were used to generate average ERPs across sentence repetitions at the character level reflecting the neural activation in the initial phase of character writing. Eight ERPs were obtained corresponding to the eight characters per sentence. The ERP epoch ranged from −300 to 700 ms relative to the first pen‐down events (time zero). A baseline correction was performed using the time window from −300 to −100 ms prior to the pen‐down events. Given that there have been no studies that utilized fast‐paced motor task paradigms to characterize the BP, the selection of the baseline window and epoch window for characterizing neural activity prior to writing each character was a heuristic decision based on the specific patterns of the current data. The major principle we used here was that the selected baseline and epoch windows should not overlap with the motor production of the previous stroke writing action. Given that the temporal interval before writing a character and the stroke in the previous character is only 300 ms and a major deflection was found at the time point of around 100 ms before the action onset, we decided to use the initial phase (−300 to −100 ms) of the interval as the baseline to characterize the preparation‐related activity before the action onset. The pen‐down event is considered as a motor event, not a stimulus; because motor events are preceded by motor preparation‐related neural activity (Kornhuber & Deecke, 1965; Leuthold & Schröter, 2011; Shibasaki et al., 1980; Xu et al., 2015), the baseline window was set to −100 ms relative to movement onset, leaving 200 ms for the regular baseline correction in ERP analysis. Note that the 100 ms pre‐movement interval is considerably shorter than the usual interval for examining the BP component in discrete, long‐interval, voluntary motor tasks where movement‐related potentials already appear at −500 ms or earlier. However, the design of the current task does not afford such long pre‐motor intervals because writing of the previous character finished only around −300 ms before the start of the character in question.

The eight ERPs were grouped into two groups of character complexity, adopting the most commonly used measure to quantify the complexity of Chinese characters – the total number of strokes composing the character (Liversedge et al., 2014; Ma & Li, 2015; Wang et al., 2014). Therefore, relatively simple characters are ‘下’, ‘书’, ‘见’ and ‘后’, whereas relatively complex characters are ‘图’, ‘前’, ‘课’ and ‘馆’. Average ERPs for simple and complex characters were compared. Given that ‘下’ is the start of the sentence which may contain substantially different neural activity than other characters due to the transition process from a long‐duration static state to the initiation of a continuous movement, we excluded ‘下’ when plotting the average ERPs for simple characters.

For ERP components with bipolar scalp topographies, dipole fitting was performed to estimate the location and moment of the dipole model that explains the data, using the DIPFIT plugin of EEGLAB. First, the template of Spherical Four‐Shell (BESA) was selected as the head model. Then, a single dipole was fitted using the fine fit option. A single dipole was selected because we only applied dipole fitting to topographies with a visual dipole (see Result section). The goodness‐of‐fit of the dipole model was assessed using the residual variance.

#### Neural activation pattern synchronized to the first character in a sentence

In addition to the ERPs locked to the onsets of each character, we also plotted the ERPs locked to the first character in a sentence, that is, to the first pen‐down event of writing the first character ‘下’ in each sentence. Because this motor action is self‐determined and not immediately preceded by other motor actions, we expected a traditional BP during the pre‐action interval. Here, the ERP epoch ranged from −700 ms to 1000 ms with respect to the first pen‐down action and was baselined at the window −700 to −500 ms. The topography of the pre‐sentence BP was calculated from the average ERP from −500 ms to 0 ms, following the conventional way of characterizing BP.

#### Analysing the effects of character complexity

To examine the effects of character complexity on the handwriting behaviour and the associated neural activations, we applied two Linear Mixed Models (LMM) with the same independent variables but with different dependent variables. In both models, the key independent variable was character complexity with two levels (see above). To control for some of the confounding effects, we introduced three control variables as described below.

The first LMM examined the effect of character complexity on behavioural data. The behavioural variable of interest is the inter‐character interval (ICI), that is, preparation time before writing each character, which was defined as the interval between the start of writing the first stroke of the current character and the end of writing the last stroke of the preceding character. We expected that characters with higher complexity would be preceded by longer ICIs than low‐complexity characters.

The second LMM examined the effect of character complexity on the amplitudes of the ERP components associated with the first point of writing a character, measured as the average amplitude at a given electrode within a time window. We tested the complexity effect on two ERP components with distinct and clearly structured spatiotemporal patterns. The first one is centred at Fz within the time window of −100 to 0 ms, assumed to be associated with preparatory neural activity for the handwriting movement. The second one is centred at Pz within the time window of 200–300 ms, capturing the major activity after the start of the writing a character. The two electrodes were selected from the epicentres of neural activity clusters. And the two time windows were selected from the time range that covers the main activation pattern in the ERP waveforms. Although the effects were only tested on these two components, the descriptive patterns of the complexity effect on ERP in general, e.g., the distribution over the scalp across different time periods, were characterized by the LMM to provide information regarding the spatiotemporal patterns of complexity effects on ERP amplitude but without the intention to test statistical significance.

In addition, three control factors were specified as fixed effects in the two models above. The first control factor was inter‐character spatial distance, which was defined as the Euclidean distance between the start of the current character and the end of the preceding character. This distance was assumed to directly affect ICI because the movement from the end of the previous character to the start of the current character is physically determined by the distance between these two points. Since the first character (‘下’) does not have a preceding character, it was excluded from the model. The second control factor was the ordinal number of the written character (e.g., ‘课’ was 2nd; ‘书’ was 5th) within a trial. The third factor was the ordinal number of the current trial within the 10 sentence repetitions. This control variable was based on the consideration that there may be practice effects over sentence repetitions. Participant identity was specified as random effect on the model's intercept.

The LMM was performed using *lme4* (Bates et al., 2015) and *lmerTest* (Kuznetsova et al., 2017) packages in R (RCoreTeam, 2020) through R Studio. The two models specified in the *lmer* were as follows:

LMM testing effects on behaviour:

*ICI ~* 1 *+ charDistance + charOrder + trialOrder + charComplexity + (1 + complexityLevel | parti)*

LMM testing effects on ERPs:

*ERP Amplitude ~* 1 *+ charDistance + charOrder + trialOrder + charComplexity + (1 + complexityLevel | parti)*

## RESULTS

### Basic kinematic characteristics of handwriting

Table 1 summarizes the descriptive statistics of the basic kinematic characteristics for writing each of the eight characters in the sentence ‘下课后图书馆前见’ (‘See you after class in front of the library’). The differences between the simple and complex characters (下, 后, 书, 见 vs. 课, 图, 馆, 前) were significant in all kinematic aspects, as supported by paired *t‐* tests: actual stroke number: *t*(100) = −75.40, *p* < .001; length: *t*(100) = −33.69, *p* < .001; duration: *t*(100) = −56.91, *p* < .001; max force of first stroke: *t*(100) = 9.15, *p* < .001; mean velocity of first stroke: *t*(100) = 11.99, *p* < .001; mean velocity while writing the entire character: *t*(100) = 23.41, *p* < .001.

**TABLE 1:** Mean and standard deviations of the kinematic characteristics for each character and for the simple and complex character groups.

Character	Simple character group					Complex character group				
	下	书	见	后	Avg.	图	前	课	馆	Avg.
Standard stroke number	3	4	4	6	4.25	8	9	10	11	9.50
Actual stroke number	2.94 (0.18)	3.77 (0.39)	3.82 (0.33)	5.11 (0.74)	3.91 (0.31)	7.51 (0.62)	7.99 (1.06)	9.62 (0.71)	9.47 (1.40)	8.66 (0.86)
Length (pixel)	292.85 (75.87)	547.33 (148.50)	503.25 (140.65)	447.91 (111.63)	448.30 (112.81)	765.57 (202.54)	692.95 (187.12)	830.07 (212.07)	799.10 (217.52)	779.69 (203.83)
Duration (ms)	771.35 (155.99)	1263.62 (227.43)	1216.03 (221.38)	1401.05 (249.19)	1166.81 (203.37)	2259.08 (350.75)	2230.99 (365.85)	2754.83 (429.64)	2933.96 (578.93)	2567.08 (433.52)
Max force of first stroke	0.53 (0.14)	0.61 (0.15)	0.56 (0.14)	0.56 (0.13)	0.57 (0.13)	0.57 (0.15)	0.50 (0.11)	0.48 (0.13)	0.53 (0.13)	0.52 (0.12)
Mean velocity of the first stroke (pixel/ms)	0.58 (0.18)	0.51 (0.18)	0.52 (0.17)	0.57 (0.18)	0.55 (0.17)	0.70 (0.23)	0.33 (0.12)	0.27 (0.10)	0.51 (0.17)	0.46 (0.15)
Mean velocity during character writing (pixel/ms)	0.39 (0.12)	0.45 (0.15)	0.43 (0.14)	0.33 (0.10)	0.40 (0.12)	0.35 (0.10)	0.32 (0.09)	0.31 (0.09)	0.28 (0.09)	0.32 (0.09)

*Note*: Standard stroke number: total number of standard strokes in the character. Actual stroke number: average number of strokes in actual writing (sometimes separate strokes were connected into single ones). Length: total length of the trajectories of the written characters. Duration: average time taken to write the entire character; Max force of the first stroke: maximal pressure of touch when writing the first stroke of the character and values are normalized to a range between 0 (no touch) and 1 (full touch). Mean velocity of the first stroke: the average velocity of writing the first stroke within a character. Mean velocity during character writing: the average velocity of writing all strokes within a character.

### Neural activation associated with the start of character writing

**FIGURE 2:**
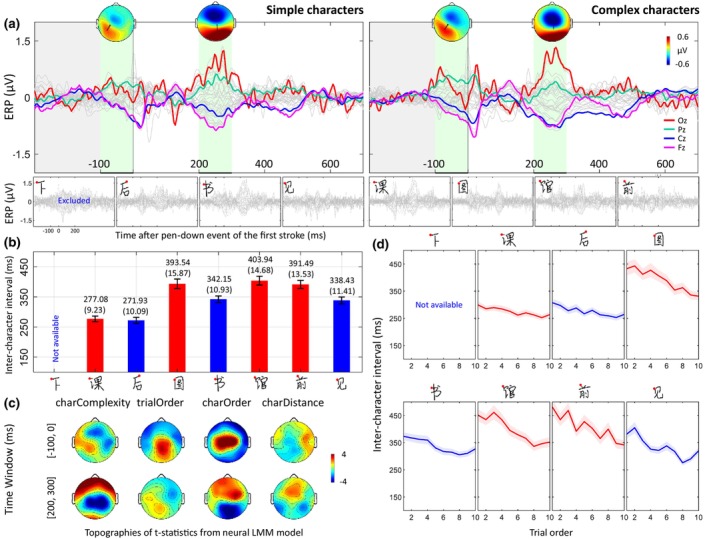
Neural and behavioural dependent variables as a function of character complexity. (a) Top: Average ERP patterns for simple and complex characters and their topographies in selected time windows (marked in green shading) −100 to 0 and 200 to 300 ms. The dipole fitted for each topography was overlaid (black arrow). The baseline window is marked in grey. Bottom: ERP patterns associated with each character. The red dots on the characters denote the first point of writing these characters in a standard way (for a larger version, please see Figure 1). (b) Means and standard errors of ICIs for each character (blue and red for simple and complex characters, respectively). ICIs for the first character (下) were not available as there is no preceding character. (c) Topographies of *t*‐statistics obtained from the fitting results of neural LMM model examining the effects of different covariates on ERP amplitudes in the time windows −100 to 0 ms (top) and 200 to 300 ms (bottom). (d) Means and standard errors of the mean of ICIs for each character across the 10 repetition trials.

Figure 2a shows the average ERP waveshapes for the simple and complex character groups, together with the topographies during the time windows −100 to 0 and 200 to 300 ms. The overall temporal patterns of ERPs of the simple and complex characters are similar, indicating good consistency of the neural activation associated with the start of character writing despite the rather small overall amplitudes of the ERPs. Importantly, there are visually discernable amplitude differences between the two levels of complexity.

In the interval immediately preceding the first pen‐down events [−100 to 0 ms] (Figure 2a) in writing the characters, the neural activities of both simple and complex character groups displayed a clear centro‐frontal negativity accompanied by a left‐parietal positivity. The centro‐frontal negativity resembles the BP but has a more anterior distribution on the scalp and much shorter time interval than what is usually reported in trials with long IRIs (e.g., Di Russo et al., 2017), and its magnitude is larger before complex than simple characters (Figure 2a). The left‐lateralized parietal positivity combined with the frontal negativity is suggestive of a tangential dipole source located in the left hemisphere. Therefore, we fitted the ERP during the pre‐onset interval with a single equivalent dipole in each condition. Indeed, the fitted dipoles were located in the left motor cortex contralateral to the writing (right) hand. The residual variances of the fit were 21% for the simple condition and 8% for the complex condition. For comparison, the residual variance of single dipole fitting to random values of 32 electrodes was around 70% on average for the present setting. The locations of the dipoles match the dipole locations fitted for the lateralized readiness potential (Hervault et al., 2021).

**FIGURE 3:**
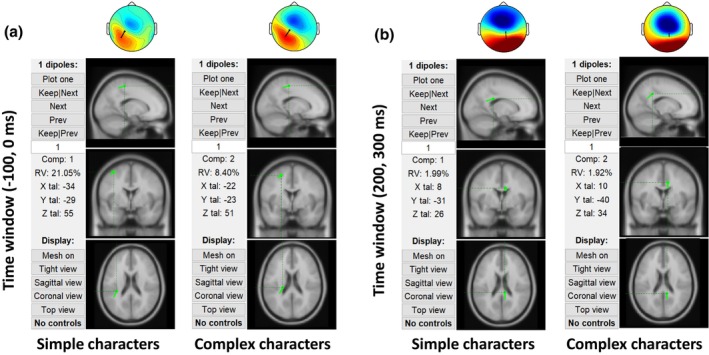
Dipole fitting results of the discovered components from the pre‐event [−100, 0 ms] and post‐event [200, 300 ms] time windows.

In the post‐event time window of 200 to 300 ms, both character groups also exhibit a bipolar scalp pattern composed of a negative frontal and a positive posterior cluster. Although the patterns are similar, the frontal cluster appears to be spatially more focused and stronger in the complex than in the simple condition. Dipole fitting was also applied on these two scalp maps, and the results together with the pre‐time window maps were shown in Figure 3. The residual variances of the fitting were 2% for both the simple and complex conditions.

### Neural activation associated with the start of sentence writing

**FIGURE 4:**
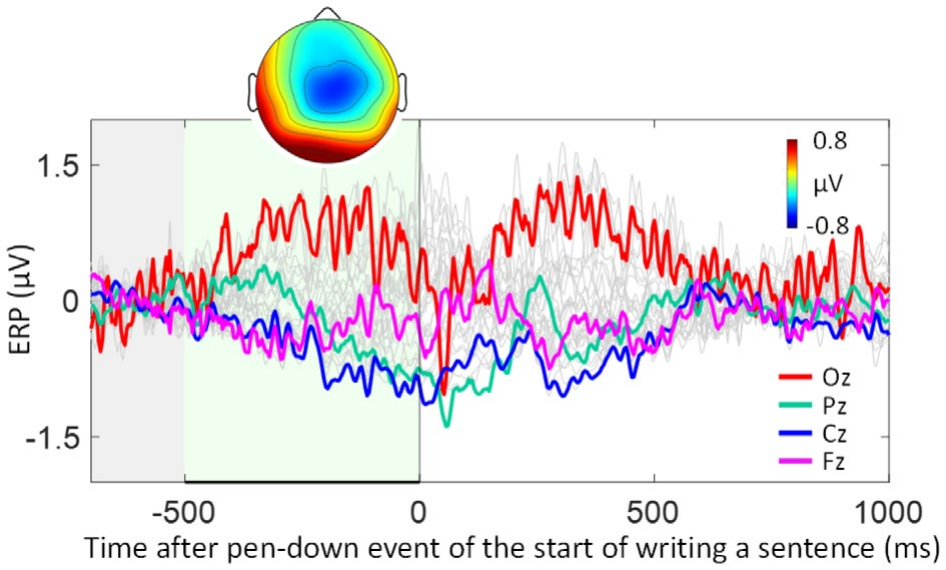
Grand average ERP patterns time‐locked to the start of sentence writing. The topography was averaged from −500 to 0 ms (marked in green). The baseline time window is marked in grey. ERP patterns from Oz, Pz, Cz and Fz electrodes are highlighted in colours.

To compare the pre‐character BP‐like frontal negativity during handwriting with a more standard BP, we plotted the average ERPs synchronized to the first pen‐down event of the first character in the sentence and extracted the topography averaged during the time window of [−500 ms, 0 ms] (see Figure 4). This BP component is not immediately preceded by other motor actions and thus should resemble a standard BP. Indeed, as shown in Figure 4, a slow negative‐going activity centred at Cz starts from around 500 ms before the onset of sentence writing. Both the time course and topography of this component resemble the classic BP (see Figure 4). Thus, the standard BP is accessible from the current experimental setting, and this BP is slightly different from the ‘online’ BP embedded in the compacted actions during handwriting. This difference will be discussed later.

### The effect of character complexity on ICI

We hypothesized that more preparation is needed before writing a character with more strokes because the motor programme for complex characters should contain more information that requires more time to be retrieved from long‐term memory. Therefore, we expected longer ICI for complex as compared to simple characters. This expectation was descriptively in line with the results shown in Figure 2b.

The naturalistic handwriting task used here required continuous writing of the multiple characters in a sentence. In such cases, the observed ICI of writing a character may be confounded by (1) the Euclidian distance between the end of the preceding character and the start of the current character, (2) the order of characters within the sentence and (3) practice effects across consecutive sentences (as shown in Figure 2d). Therefore, we included these three potentially confounding factors, together with character complexity, as fixed effects in the linear mixed model to test the effect of character complexity on ICI. As shown in Table 2, the results confirm that character complexity has a significant effect on ICIs even after regressing out the (significant) effects of the confounding factors. Briefly, characters that comprise more strokes are associated with longer ICIs even when the confounding factors are controlled for.

**TABLE 2:** Fixed effects on ICIs estimated using the linear mixed model (LMM).

	*b*	*SE*	CI (95%)		*df*	*t*
			Low	High		
(Intercept)	202.03	19.26	164.27	239.78	766.18	10.49^***^
charComplexity	76.16	9.75	57.04	95.28	108.08	7.81^***^
charDistance	0.83	0.10	0.64	1.02	5696.83	8.54^***^
charOrder	18.57	1.67	15.30	21.84	6444.26	11.12^***^
trialOrder	−9.77	1.12	−11.97	−7.57	6418.44	−8.70^***^

Abbreviations: *b*, co‐efficient in the linear mixed model; CI, confidence interval; *SE*: standard error; *t*: *t*‐statistics.

*** *p* < .001.

### The effects of character complexity on ERP amplitudes

As our central aim, we examined the effect of character complexity on the ERP amplitudes associated with the initial points of character writing. If character complexity affects the cognitive processes involved in handwriting, for example, motor programme retrieval and action preparation, it should be reflected in ERPs before writing onsets. Similar to the behavioural analysis, we first applied LMMs to the ERP amplitudes averaged within the time window before writing onset (i.e. −100 to 0 ms). Again, the confounding factors of character distance, character order and trial order were included in the LMMs as independent variables (see Method). According to previous findings on the relationship between BP amplitude and response complexity based on discrete action paradigms, we assumed that writing more complex characters should induce a larger negative potential resembling BP due to proportionally more complex pattern retrieval and motor planning (Xu et al., 2015). This relationship should hold even when the time period for the preparation (here only 100 ms) in compact naturalistic motor task is much shorter than in discrete motor tasks. As expected, character complexity induced a significant negative‐going ERP effect in the frontal region at Fz (charComplexity: *t*(3574.28) = −2.47, *p* = .014; charDistance: *t*(2465.15) = −0.21, *p* = .832; charOrder: *t*(6568.04) = 1.34, *p* = .179; trialOrder: *t*(6524.36) = −0.22, *p* = .829 from LMM results) in the pre‐event time window [−100 to 0 ms] (see Figure 2c, top left). This result is consistent with the descriptive result shown in Figure 2a.

In addition, we examined the neural effect of character complexity in the post‐event interval (i.e. 200 to 300 ms). The underlying assumption is that writing complex characters with more strokes should be more cognitively loaded during the writing process, which may suppress event‐evoked ERP components (Ghani et al., 2020). After regressing out the confounding factors, the LMM results on the post‐event interval support our assumption, showing that character complexity does have significant effects on multiple electrodes mainly located in the parietal regions (see Figure 2c, bottom left). Higher character complexity appears to induce a negativity or suppress a positivity in parietal regions centred at Pz (charComplexity: *t*(116.12) = −3.40, *p* < .001; charDistance: *t*(3276.08) = −0.12, *p* = .902; charOrder: *t*(6484.44) = −3.71, *p* < .001; trialOrder: *t*(6428.86) = 1.56, *p* = .119 from LMM results).

## DISCUSSION

In this work, we asked whether we can identify specific neural dynamic activities indicative of real‐time cognitive variables associated with the complicated and naturalistic task of self‐paced handwriting. More specifically, we used handwriting of Chinese script as a venue to examine whether the complexity in motor programme content (operationalized as the complexity of Chinese characters) modulates preparation and response‐related neural activities – a question that is usually examined in discrete motor paradigms. The present study is one of the first to investigate related neural effects in a naturalistic paradigm where many processes and factors are intermingled. As such, there are several factors confounding the effects of complexity. Such confounding issues might impose inherent limitations on many naturalistic paradigms. Here, we identified and controlled three confounding factors by including them in the same LMM model and showed that character complexity contributes a unique share of variance to ICIs and ERP amplitudes in writing Chinese characters. In the following, we discussed the major findings, implications and limitations in this work.

### Behavioural effect of character complexity

The first major finding of the present study is the significant effect of character complexity on ICI, that is, ICIs preceding more complex characters are longer. This result is in line with the assumption that more complex characters require more time to retrieve morphology and for subsequent motor programming before motor execution. It is important to note that the observed effect had excluded the contributions of the major confounding factors, that is, Euclidian distance between the end of the previous and the start of the current character, sequential order of the character and practice effects of repetitively writing the same sentence.

The complexity effect on ICIs is consistent with previous findings based on separate and elementary motor action paradigms showing longer RTs for more complex motor sequences (e.g., finger tapping, button/key pressing; Canic & Franks, 1989; De Kleine & Van der Lubbe, 2011; Dhamala et al., 2003). In these paradigms, participants usually need to perform motor responses with different levels of complexity following a response signal under different experimental conditions. The RT measured as the interval between cue presentation and movement initiation reflects the motor preparation process; for instance, the more parameters of a movement are specified in the cue, the longer is RT (Rosenbaum, 1980). In our task, characters with higher complexity (more strokes) can be viewed as initiating more complex motor preparation because complex characters contain more parameters in shape, stroke orientation, overall layout and inter‐stroke spatial relationships than simple characters (Chen & Kao, 2002). In this sense, longer ICIs were expected for complex characters that were initiated on a self‐paced basis. Another unique aspect of the present experiment is that the actions were not triggered by external cues but were self‐initiated by the participants.

It is worth noting that the ICI between adjacent characters in handwriting is not exactly equivalent to the RT measured between the onset of a response signal and the cue triggering a motor response in discrete motor tasks. During continuous and naturalistic handwriting production, participants may simultaneously verify the accuracy of the previous character they have just written while retrieving the motor programme for the next character and moving their hand accordingly to initiate writing the next character. Therefore, the effect on ICI could be due to the evaluation rather than movement initiation processes. To examine this confounding issue, we conducted additional analysis by including the character complexity (assuming that complexity affects post‐writing evaluation) of the previous character into the LMMs. The result still showed a significant next‐character complexity effect at the behavioural level (*t* = 2.67, *p* = .008). This additional result further supports that the ICIs are significantly affected by the motor preparation for writing the next character during continuous handwriting production. However, because the complexity of current and previous characters in the current limited data is not very well controlled in its randomness, we did not replace the main statistical analysis in the Result section by introducing the complexity of previous characters.

It is also worthwhile to note that the effect of character complexity may be confounded by the practice effect caused by the repetitive handwriting of the same sentence 10 times. This is because the frequent practice within a short period may improve handwriting fluency, making a complex character less challenging as the task progresses. To further control this confounding effect, we conducted another analysis by introducing the interaction effect between practice times (i.e. trialOrder) and character complexity into the LMMs. The result still showed a significant character complexity effect at the behavioural level (*t* = 5.74, *p* < .001), and no significant interaction effect was found between practice times and character complexity (*t* = −1.19, *p* = .234). This result further supports that the ICIs are significantly affected by the character complexity even after multiple repetitions. However, we did not replace the main statistical analysis in the Result section as the new model could not resolve random effects on the neural data due to the limited data in this study.

### BP in naturalistic motor tasks

The second major finding concerns the appearance of BP in the highly compact online motor task. BP is a classic motor‐related ERP component that has been widely interpreted as a neural representation of movement preparation. However, as yet, the component's identification is based on discrete action paradigms with long interaction interval (see Olsen et al., 2021, for a review). Whether or not a BP can be identified in highly skilled and compact action sequences tasks, such as handwriting, typing, or playing an instrument, is unknown. In this study, we identified a clear frontal negative‐going component immediately preceding the action of writing an individual character. Although this component is confined to a much shorter time period (related to the present compact motor action task), it largely resembles the classic BP in its time course, functional association with motor complexity and scalp topography (Schurger et al., 2021; Shibasaki & Hallett, 2006). For comparison, we plotted the BP preceding the first motor action in a sentence (see Figure 4), which shows a standard pattern. Compared with this standard BP, the online character‐level BP (henceforth termed online BP) also showed a frontal negative‐going drift over time preceding the action. Most importantly, the magnitude of the online BP was more pronounced in preparation of more complex characters, in line with the literature about the relationships between the standard BP and motor complexity (Leuthold & Schröter, 2011; Mussini et al., 2021).

The resemblance of the online BP and the standard BP suggests the existence of a BP in compact actions, reported in the present work for the first time. Nevertheless, the online BP differs from the standard BP in discrete motor task in three respects, first, in its shorter duration, as mentioned. The second difference is the slightly more frontal scalp topography of the online BP as compared to the typical BP (as shown in our pre‐sentence BP). The third difference is the left parietal positivity which makes the online BP look like resulting from a dipolar neural generator. The second and third differences may be due to the feature of the present task that the motor programming‐related components may dominate over motor preparation‐related activity (Ulrich et al., 1998). Thus, the online BP may reflect a lateralized readiness potential (LRP) for the right hand (Vidal et al., 2003) – the only hand used here.

Taken together, the results above, for the first time, demonstrate the technical feasibility of extracting a BP from fast‐paced naturalistic action paradigm, albeit with clear but expected variation from the conventional BP from the discrete motor paradigms. And given the functional role of BP, it also suggests that complex fluent motor production with close‐to‐continuous movement pattern can be broken down into basic unit of action preparation, programming and execution from which cognitive effects (such as complexity) can be studied.

### Neural effect of character complexity

The third major finding of this study is the significant effect of character complexity on the neural activity associated with writing individual characters, more specifically, on the ERP patterns associated with the initial point of writing each character.

First, as mentioned above, we found that character complexity has a significant effect on the amplitude of the online BP in a way that is consistent with the previously reported effect of motor complexity on BP, that is, larger negativity is associated with more complex motor demand or complexity (Cui et al., 2000; De Kleine & Van der Lubbe, 2011; Hackley & Miller, 1995; Kitamura et al., 1993; Leuthold & Schröter, 2011; Prescoot, 1986). This effect could be linked to different load of motor preparation before writing characters with different complexities, including retrieval of visuo‐kinesthetic and sequential motor engrams (Planton et al., 2013). This finding further supports the BP‐ or LRP‐like nature of the centro‐frontal negativity found in this study, even though it is a ‘shrunken’ version. It is noted that this effect remains significant even after introducing the complexity of the previous character as an additional independent variable (*t* = −3.13, *p* = .002).

The second complexity effect on neural activity was observed in the post‐event evoked component and appeared to have an amplitude‐suppressing effect for the positive‐going component with a primary effect on the central parietal region (see Figure 2). Since this paradigm is relatively new and there are few comparable paradigms in the literature, we can only speculate about the post‐event effect of complexity. Our first suggestion is that the effect could be linked to the N400 component. It has been reported that word frequency and orthographic neighbourhood size (Hauk & Pulvermüller, 2004) in English are associated with N400 amplitude. Notably, in a visual stimulus‐based ERP study of Chinese words (Tsang & Zou, 2022), it was found that a higher number of strokes is associated with a smaller ERP amplitude in the parietal regions starting from around 400 ms. This finding is in line with our results despite the timing difference, which is plausible because our task was not based on visual stimulus presentation and might show shorter latency in this component. It is noted that the effect remained significant even after introducing the complexity of the previous character as an additional independent variable (*t* = −2.45, *p* = .015).

Given the possible confound by the linguistic features of the characters, we further explored whether the introduction of major linguistic factor would substantially change the results. Therefore, we added one representative linguistic feature, the frequency of use of the characters (which should conceptually influence the motor programme retrieval) into the LMM. The frequencies of use for the eight characters in our experiment were obtained from SUBTLEX‐CH, a corpus of Chinese character/word frequencies derived from film subtitles (Cai & Brysbaert, 2010). After adding this variable into the LMMs, we found that the character complexity effect on the post‐event component at Pz electrode became even more significant (*t* = −4.25, *p* < .001). This further corroborated the unique role of character complexity in modulating the post‐event negativity. However, because of the limited number of characters and the danger of over‐complicating the model, we refrained from systematically examining all linguistic features in the current study (see discussion on limitations below). Hence, because we did not control other linguistic aspect of the characters, it remains inconclusive if the negative effect we found here is an N400‐like component, which may be investigated in the future.

Another potential interpretation of the late complexity effect is due to the higher cognitive load associated with more complex characters, which may suppress the majority of sensory ERP components after the events (Ghani et al., 2020). In this case, the negativity in the difference topography (complex minus simple) could be from a lower amplitude of the P3 component for more complex characters. However, this interpretation is based on the assumption that the P3 is an ERP component not directly belonging to character processing (and thus was suppressed). If the P3 is part of the character processing activity, it should be expected to be enhanced because many literatures have reported increased P3 due to task complexity (Polich, 1987, 2007). Future research with more systematic control of linguistic factors may be able to determine the nature of the late component in the present paradigm.

### Limitations

The main limitation of the present study is that the number of characters was rather small and consequently, the diversity of characters was low. This makes the task less naturalistic because in reality people usually write contents with a high diversity of characters. A related limitation is the high repetitiveness of the handwriting activity. Due to the low number of characters, the participants were required to repetitively write the same sentence 10 times. It is conceivable that the handwriting activity at the later phase became increasingly artificial due to the practice effect. The low number of characters also made us refrain from (1) adding more linguistic features of the characters into the LMM analysis and (2) investigating the impact of practice by controlling for the interaction effect between repetitions and character complexity in the LMM analysis. To address these issues, further research should collect more different characters in a way that is controlled in terms of linguistic features (e.g., commonality of composing radicals, imageability and participants' perceived familiarity), which would allow for a more systematic description of the complexity effect and make the task more naturalistic. Finally, some technical aspects of the present study could still be improved. For example, writing on an electronic tablet is technically different from the genuine process of writing on paper, even though we have put on a paper‐feel screen protector on the tablet screen. This could be improved by establishing a synchronization system between EEG and a handwriting recording system based on real paper.

## CONCLUSION AND IMPLICATIONS FOR FUTURE RESEARCH

This study advanced our understanding of the dynamic pattern of handwriting‐related neural activities and its relationship with the complexity of characters to be written. Moreover, we demonstrated the feasibility of studying neurocognitive processes associated with motor production in complex naturalistic contexts. Thence, we provided the first evidence showing that neural dynamics underlying naturalistic continuous motor production are similar to discrete motor production processes in the exhibition of BP component albeit with quantitative differences in timing and magnitude. This result strongly indicates the feasibility of further studies on the neural correlates of complex motor production in naturalistic situations, especially writing, its acquisition and disorders.

As for implications for future research, one potential direction is to further explore whether the gender factor affects the effect of character complexity on the naturalistic Chinese handwriting process, which may inform the handwriting education on young children. Although our additional analyses did not show any significant effect of gender at both behavioural (*t* = 0.63, *p* = .716) and neural levels (Fz at pre‐event time window: *t* = −0.96, *p* = .337; Pz at post‐event time window: *t* = −1.52, *p* = .130), the gender difference in handwriting has been pervasively found in previous research studies (Maurer, 2024; Yang et al., 2020) and may manifest in other aspects of behavioural or neural characteristics that we did not examine in the current work. Another further direction is to apply deconvolution methods (e.g., Ehinger & Dimigen, 2019) to address the inter‐event overlapping issues and further confirm the neural activation time course associated with the elementary motor actions in highly compacted naturalistic tasks. The third direction is to manipulate different stages in the handwriting activity and examine how neural dynamic activity changes, thereby testing different cognitive models of handwriting. The fourth direction is to collect and examine neural effects from naturalistic tasks and use them as indicators of learning, skill acquisition, development and disorders to inform intervention research in handwriting education.

## AUTHOR CONTRIBUTIONS

**Leisi Pei:** Conceptualization; investigation; methodology; software; data curation; formal analysis; visualization; writing – original draft; writing – review and editing. **Werner Sommer:** Conceptualization; writing – review and editing; methodology. **Guang Ouyang:** Conceptualization; methodology; resources; writing – review and editing; funding acquisition.

## FUNDING INFORMATION

This work was supported by the Hong Kong Research Grant Council (17609321) and Seed Fund for Basic Research from the University of Hong Kong (2302101550).

## CONFLICT OF INTEREST STATEMENT

The authors declare no competing interests.

## Data Availability

The datasets used and analysed during the current study are available from the corresponding author on reasonable request.

## References

[bjop12742-bib-0001] Askvik, E. O. , Weel, F. , & Meer, A. (2020). The importance of cursive handwriting over typewriting for learning in the classroom: A high‐density EEG study of 12‐year‐old children and Young adults. Frontiers in Psychology, 11, e01810. 10.3389/fpsyg.2020.01810 PMC7399101 32849069

[bjop12742-bib-0002] Bates, D. , Mächler, M. , Bolker, B. M. , & Walker, S. C. (2015). Fitting linear mixed‐effects models using lme4. Journal of Statistical Software, 67(1), 1–48. 10.18637/jss.v067.i01

[bjop12742-bib-0003] Cai, Q. , & Brysbaert, M. (2010). SUBTLEX‐CH: Chinese word and character frequencies based on film subtitles. PLoS One, 5(6), e10729. 10.1371/journal.pone.0010729 20532192 PMC2880003

[bjop12742-bib-0004] Canic, M. J. , & Franks, I. M. (1989). Response preparation and latency in patterns of tapping movements. Human Movement Science, 8(2), 123–139. 10.1016/0167-9457(89)90013-4

[bjop12742-bib-0005] Chen, J.‐Y. , & Cherng, R.‐J. (2013). The proximate unit in Chinese handwritten character production. Frontiers in Psychology, 4, e00517. 10.3389/fpsyg.2013.00517 PMC3738862 23950752

[bjop12742-bib-0006] Chen, X. , & Kao, H. S. R. (2002). Visual‐spatial properties and orthographic processing of Chinese characters. In Cognitive neuroscience studies of the Chinese language (pp. 175–194). Hong Kong University Press.

[bjop12742-bib-0007] Cui, R. Q. , Egkher, A. , Huter, D. , Lang, W. , Lindinger, G. , & Deecke, L. (2000). High resolution spatiotemporal analysis of the contingent negative variation in simple or complex motor tasks and a non‐motor task. Clinical Neurophysiology, 111(10), 1847–1859. 10.1016/S1388-2457(00)00388-6 11018502

[bjop12742-bib-0008] De Kleine, E. , & Van der Lubbe, R. H. J. (2011). Decreased load on general motor preparation and visual‐working memory while preparing familiar as compared to unfamiliar movement sequences. Brain and Cognition, 75(2), 126–134. 10.1016/j.bandc.2010.10.013 21094573

[bjop12742-bib-0009] Delorme, A. , & Makeig, S. (2004). EEGLAB: An open source toolbox for analysis of single‐trial EEG dynamics including independent component analysis. Journal of Neuroscience Methods, 134(1), 9–21. 15102499 10.1016/j.jneumeth.2003.10.009

[bjop12742-bib-0010] Dhamala, M. , Pagnoni, G. , Wiesenfeld, K. , Zink, C. F. , Martin, M. , & Berns, G. S. (2003). Neural correlates of the complexity of rhythmic finger tapping. NeuroImage, 20(2), 918–926. 10.1016/S1053-8119(03)00304-5 14568462

[bjop12742-bib-0011] Di Russo, F. , Berchicci, M. , Bozzacchi, C. , Perri, R. L. , Pitzalis, S. , & Spinelli, D. (2017). Beyond the “Bereitschaftspotential”: Action preparation behind cognitive functions. Neuroscience & Biobehavioral Reviews, 78, 57–81. 10.1016/j.neubiorev.2017.04.019 28445742

[bjop12742-bib-0012] Doyon, J. , Gabitov, E. , Vahdat, S. , Lungu, O. , & Boutin, A. (2018). Current issues related to motor sequence learning in humans. Current Opinion in Behavioral Sciences, 20, 89–97. 10.1016/j.cobeha.2017.11.012

[bjop12742-bib-0013] Ehinger, B. V. , & Dimigen, O. (2019). Unfold: An integrated toolbox for overlap correction, non‐linear modeling, and regression‐based EEG analysis. PeerJ, 7, e7838. 10.7717/peerj.7838 31660265 PMC6815663

[bjop12742-bib-0014] Ghani, U. , Signal, N. , Niazi, I. K. , & Taylor, D. (2020). ERP based measures of cognitive workload: A review. Neuroscience & Biobehavioral Reviews, 118, 18–26. 32707343 10.1016/j.neubiorev.2020.07.020

[bjop12742-bib-0015] Graham, S. , & Weintraub, N. (1996). A review of handwriting research: Progress and prospects from 1980 to 1994. Educational Psychology Review, 8(1), 7–87. 10.1007/BF01761831

[bjop12742-bib-0016] Hackley, S. A. , & Miller, J. (1995). Response complexity and precue interval effects on the lateralized readiness potential. Psychophysiology, 32(3), 230–241. 10.1111/j.1469-8986.1995.tb02952.x 7784531

[bjop12742-bib-0017] Hauk, O. , & Pulvermüller, F. (2004). Effects of word length and frequency on the human event‐related potential. Clinical Neurophysiology, 115(5), 1090–1103. 10.1016/j.clinph.2003.12.020 15066535

[bjop12742-bib-0018] Henry, F. M. , & Rogers, D. E. (1960). Increased response latency for complicated movements and a “memory drum” theory of Neuromotor reaction. Research Quarterly of the American Association for Health, Physical Education, and Recreation, 31(3), 448–458. 10.1080/10671188.1960.10762052

[bjop12742-bib-0019] Hervault, M. , Zanone, P.‐G. , Buisson, J.‐C. , & Huys, R. (2021). Cortical sensorimotor activity in the execution and suppression of discrete and rhythmic movements. Scientific Reports, 11(1), 22364. 10.1038/s41598-021-01368-2 34785710 PMC8595306

[bjop12742-bib-0020] Hulstijn, W. , & van Galen, G. P. (1983). Programming in handwriting: Reaction time and movement time as a function of sequence length. Acta Psychologica, 54(1), 23–49. 10.1016/0001-6918(83)90021-5

[bjop12742-bib-0021] Hulstijn, W. , & van Galen, G. P. (1988). Levels of motor programming in writing familiar and unfamiliar symbols. In A. M. Colley & J. R. Beech (Eds.), Advances in psychology (Vol. 55, pp. 65–85). Elsevier.

[bjop12742-bib-0022] Jahanshahi, M. (2003). Chapter 15 Reaction time as an index of motor preparation/programming and speed of response initiation. In M. Hallett (Ed.), Handbook of clinical neurophysiology (Vol. 1, pp. 203–229). Elsevier.

[bjop12742-bib-0023] Katanoda, K. , Yoshikawa, K. , & Sugishita, M. (2001). A functional MRI study on the neural substrates for writing. Human Brain Mapping, 13(1), 34–42. 10.1002/hbm.1023 11284045 PMC6871921

[bjop12742-bib-0024] Kitamura, J. I. , Shibasaki, H. , & Kondo, T. (1993). A cortical slow potential is larger before an isolated movement of a single finger than simultaneous movement of two fingers. Electroencephalography and Clinical Neurophysiology, 86(4), 252–258. 10.1016/0013-4694(93)90106-6 7682928

[bjop12742-bib-0025] Klapp, S. T. , Abbott, J. , Coffman, K. , Greim, D. , Snider, R. , & Young, F. (1979). Simple and choice reaction time methods in the study of motor programming. Journal of Motor Behavior, 11(2), 91–101. 10.1080/00222895.1979.10735177 15189802

[bjop12742-bib-0026] Kornhuber, H. H. , & Deecke, L. (1965). Hirnpotentialänderungen bei Willkürbewegungen und passiven Bewegungen des Menschen: Bereitschaftspotential und reafferente Potentiale. Pflüger's Archiv für Die Gesamte Physiologie Des Menschen Und der Tiere, 284(1), 1–17. 10.1007/BF00412364 14341490

[bjop12742-bib-0027] Kuznetsova, A. , Brockhoff, P. B. , & Christensen, R. H. B. (2017). lmerTest package: Tests in linear mixed effects models. Journal of Statistical Software, 82(13), 1–26. 10.18637/jss.v082.i13

[bjop12742-bib-0028] Leuthold, H. , & Schröter, H. (2011). Motor programming of finger sequences of different complexity. Biological Psychology, 86(1), 57–64. 10.1016/j.biopsycho.2010.10.007 20955758

[bjop12742-bib-0029] Li, J. , Hong, L. , Bi, H.‐Y. , & Yang, Y. (2021). Functional brain networks underlying automatic and controlled handwriting in Chinese. Brain and Language, 219, 104962. 10.1016/j.bandl.2021.104962 33984629

[bjop12742-bib-0030] Liversedge, S. P. , Zang, C. , Zhang, M. , Bai, X. , Yan, G. , & Drieghe, D. (2014). The effect of visual complexity and word frequency on eye movements during Chinese reading. Visual Cognition, 22(3–4), 441–457. 10.1080/13506285.2014.889260

[bjop12742-bib-0031] Longcamp, M. , Lagarrigue, A. , Nazarian, B. , Roth, M. , Anton, J.‐L. , Alario, F.‐X. , & Velay, J.‐L. (2014). Functional specificity in the motor system: Evidence from coupled fMRI and kinematic recordings during letter and digit writing. Human Brain Mapping, 35(12), 6077–6087. 10.1002/hbm.22606 25093278 PMC6868974

[bjop12742-bib-0032] Ma, G. , & Li, X. (2015). How character complexity modulates eye movement control in Chinese reading. Reading and Writing, 28(6), 747–761. 10.1007/s11145-015-9548-1

[bjop12742-bib-0033] Maurer, M. N. (2024). Correlates of early handwriting: Differential patterns for girls and boys. Early Education and Development, 35(4), 843–858. 10.1080/10409289.2023.2244349

[bjop12742-bib-0034] McHale, K. , & Cermak, S. A. (1992). Fine motor activities in elementary school: Preliminary findings and provisional implications for children with fine motor problems. The American Journal of Occupational Therapy, 46(10), 898–903. 10.5014/ajot.46.10.898 1463061

[bjop12742-bib-0035] Mussini, E. , Berchicci, M. , Bianco, V. , Perri, R. L. , Quinzi, F. , & Di Russo, F. (2021). Effect of task complexity on motor and cognitive preparatory brain activities. International Journal of Psychophysiology, 159, 11–16. 10.1016/j.ijpsycho.2020.11.008 33227366

[bjop12742-bib-0036] Olsen, S. , Alder, G. , Williams, M. , Chambers, S. , Jochumsen, M. , Signal, N. , … Rashid, U. (2021). Electroencephalographic recording of the movement‐related cortical potential in ecologically valid movements: A scoping review. Frontiers in Neuroscience, 15, e721387. 10.3389/fnins.2021.721387 PMC8505671 34650399

[bjop12742-bib-0037] Palmis, S. , Danna, J. , Velay, J.‐L. , & Longcamp, M. (2017). Motor control of handwriting in the developing brain: A review. Cognitive Neuropsychology, 34(3–4), 187–204. 10.1080/02643294.2017.1367654 28891745

[bjop12742-bib-0038] Pei, L. , Longcamp, M. , Leung, F. K.‐S. , & Ouyang, G. (2021). Temporally resolved neural dynamics underlying handwriting. NeuroImage, 244, 118578. 10.1016/j.neuroimage.2021.118578 34534659

[bjop12742-bib-0039] Pivik, R. T. , Broughton, R. J. , Coppola, R. , Davidson, R. J. , Fox, N. , & Nuwer, M. R. (1993). Guidelines for the recording and quantitative analysis of electroencephalographic activity in research contexts. Psychophysiology, 30(6), 547–558. 10.1111/j.1469-8986.1993.tb02081.x 8248447

[bjop12742-bib-0040] Plamondon, R. , & Maarse, F. J. (1989). An evaluation of motor models of handwriting. IEEE Transactions on Systems, Man, and Cybernetics, 19(5), 1060–1072. 10.1109/21.44021

[bjop12742-bib-0041] Plamondon, R. , Stelmach, G. E. , & Teasdale, N. (1990). Motor program coding representation from a handwriting generator model: The production of line responses. Biological Cybernetics, 63(6), 443–451. 10.1007/BF00199576

[bjop12742-bib-0042] Planton, S. , Jucla, M. , Roux, F.‐E. , & Démonet, J.‐F. (2013). The “handwriting brain”: A meta‐analysis of neuroimaging studies of motor versus orthographic processes. Cortex, 49(10), 2772–2787. 10.1016/j.cortex.2013.05.011 23831432

[bjop12742-bib-0043] Polich, J. (1987). Task difficulty, probability, and inter‐stimulus interval as determinants of P300 from auditory stimuli. Electroencephalography and Clinical Neurophysiology/Evoked Potentials Section, 68(4), 311–320. 10.1016/0168-5597(87)90052-9 2439311

[bjop12742-bib-0044] Polich, J. (2007). Updating P300: An integrative theory of P3a and P3b. Clinical Neurophysiology, 118(10), 2128–2148. 10.1016/j.clinph.2007.04.019 17573239 PMC2715154

[bjop12742-bib-0045] Prescoot, J. (1986). The effects of response parameters on CNV amplitude. Biological Psychology, 22(2), 107–135. 10.1016/0301-0511(86)90039-6 3741934

[bjop12742-bib-0046] RCoreTeam . (2020). R: A language and environment for statistical computing. R Foundation for Statistical Computing. Retrieved from https://www.R‐project.org

[bjop12742-bib-0047] Richards, T. L. , Berninger, V. W. , Stock, P. , Altemeier, L. , Trivedi, P. , & Maravilla, K. (2009). Functional magnetic resonance imaging sequential‐finger movement activation differentiating good and poor writers. Journal of Clinical and Experimental Neuropsychology, 31(8), 967–983. 10.1080/13803390902780201 19358006 PMC2829117

[bjop12742-bib-0048] Rosenbaum, D. A. (1980). Human movement initiation: Specification of arm, direction, and extent. Journal of Experimental Psychology: General, 109, 444–474. 10.1037/0096-3445.109.4.444 6449531

[bjop12742-bib-0049] Schmitz, J. , Packheiser, J. , Birnkraut, T. , Hinz, N.‐A. , Friedrich, P. , Güntürkün, O. , & Ocklenburg, S. (2019). The neurophysiological correlates of handedness: Insights from the lateralized readiness potential. Behavioural Brain Research, 364, 114–122. 10.1016/j.bbr.2019.02.021 30768993

[bjop12742-bib-0050] Schurger, A. , Hu, P. B. , Pak, J. , & Roskies, A. L. (2021). What is the readiness potential? Trends in Cognitive Sciences, 25(7), 558–570. 10.1016/j.tics.2021.04.001 33931306 PMC8192467

[bjop12742-bib-0051] Shibasaki, H. , Barrett, G. , Halliday, E. , & Halliday, A. M. (1980). Components of the movement‐related cortical potential and their scalp topography. Electroencephalography and Clinical Neurophysiology, 49(3), 213–226. 10.1016/0013-4694(80)90216-3 6158398

[bjop12742-bib-0052] Shibasaki, H. , & Hallett, M. (2006). What is the Bereitschaftspotential? Clinical Neurophysiology, 117(11), 2341–2356. 10.1016/j.clinph.2006.04.025 16876476

[bjop12742-bib-0053] Smulders, F. T. Y. , Kok, A. , Kenemans, J. L. , & Bashore, T. R. (1995). The temporal selectivity of additive factor effects on the reaction process revealed in ERP component latencies. Acta Psychologica, 90(1), 97–109. 10.1016/0001-6918(95)00032-P 8525879

[bjop12742-bib-0054] Sternberg, S. , Knoll, R. L. , & Turock, D. L. (1990). Hierarchical control in the execution of action sequences: Tests of two invariance properties (1st ed., pp. 3–55). Psychology Press.

[bjop12742-bib-0055] Sternberg, S. , Monsell, S. , Knoll, R. L. , & Wright, C. E. (1978). 6 – The latency and duration of rapid movement sequences: Comparisons of speech and typewriting. In G. E. Stelmach (Ed.), Information processing in motor control and learning (pp. 117–152). Academic Press.

[bjop12742-bib-0056] Stief, V. , Leuthold, H. , Miller, J. , Sommer, W. , & Ulrich, R. (1998). The effect of response complexity on the lateralized readiness potential. Zeitschrift für Psychologie mit Zeitschrift für Angewandte Psychologie, 206(4), 305–319.

[bjop12742-bib-0057] Swett, B. A. , Contreras‐Vidal, J. L. , Birn, R. , & Braun, A. (2010). Neural substrates of Graphomotor sequence learning: A combined fMRI and kinematic study. Journal of Neurophysiology, 103(6), 3366–3377. 10.1152/jn.00449.2009 20375250 PMC2888252

[bjop12742-bib-0058] Teulings, H.‐L. (1996). Chapter 10 Handwriting movement control. In H. Heuer & S. W. Keele (Eds.), Handbook of perception and action (Vol. 2, pp. 561–613). Academic Press.

[bjop12742-bib-0059] Tsang, Y.‐K. , & Zou, Y. (2022). An ERP megastudy of Chinese word recognition. Psychophysiology, 59(11), e14111. 10.1111/psyp.14111 35609148

[bjop12742-bib-0060] Ulrich, R. , Leuthold, H. , & Sommer, W. (1998). Motor programming of response force and movement direction. Psychophysiology, 35(6), 721–728. 10.1111/1469-8986.3560721 9844433

[bjop12742-bib-0061] van Galen, G. P. , & Teulings, H.‐L. (1983). The independent monitoring of form and scale factors in handwriting. Acta Psychologica, 54(1), 9–22. 10.1016/0001-6918(83)90020-3

[bjop12742-bib-0062] Vidal, F. , Grapperon, J. , Bonnet, M. , & Hasbroucq, T. (2003). The nature of unilateral motor commands in between‐hand choice tasks as revealed by surface Laplacian estimation. Psychophysiology, 40(5), 796–805. 10.1111/1469-8986.00080 14696733

[bjop12742-bib-0063] Wang, H. , He, X. , & Legge, G. E. (2014). Effect of pattern complexity on the visual span for Chinese and alphabet characters. Journal of Vision, 14(8), 6. 10.1167/14.8.6 PMC4083876 24993020

[bjop12742-bib-0064] Winkler, I. , Haufe, S. , & Tangermann, M. (2011). Automatic classification of Artifactual ICA‐components for artifact removal in EEG signals. Behavioral and Brain Functions, 7(1), 30. 10.1186/1744-9081-7-30 21810266 PMC3175453

[bjop12742-bib-0065] Wymbs, N. F. , & Grafton, S. T. (2014). The human motor system supports sequence‐specific representations over multiple training‐dependent timescales. Cerebral Cortex, 25(11), 4213–4225. 10.1093/cercor/bhu144 24969473 PMC4747644

[bjop12742-bib-0066] Xiaolin, L. , & Dit‐Yan, Y. (1997). On‐line handwritten alphanumeric character recognition using dominant points in strokes. Pattern Recognition, 30(1), 31–44. 10.1016/S0031-3203(96)00052-0

[bjop12742-bib-0067] Xu, L. , Sommer, W. , & Masaki, H. (2015). The structure of motor programming: Evidence from reaction times and lateralized readiness potentials: Motor programming. Psychophysiology, 52(1), 149–155. 10.1111/psyp.12296 25082470

[bjop12742-bib-0068] Yang, Y. , Tam, F. , Graham, S. J. , Sun, G. , Li, J. , Gu, C. , … Tao, R. (2020). Men and women differ in the neural basis of handwriting. Human Brain Mapping, 41(10), 2642–2655. 10.1002/hbm.24968 32090433 PMC7294055

[bjop12742-bib-0069] Yang, Y. , Zuo, Z. , Tam, F. , Graham, S. J. , Tao, R. , Wang, N. , & Bi, H.‐Y. (2019). Brain activation and functional connectivity during Chinese writing: An fMRI study. Journal of Neurolinguistics, 51, 199–211. 10.1016/j.jneuroling.2019.03.002

[bjop12742-bib-0070] Zelaznik, H. N. , & Hahn, R. (1985). Reaction time methods in the study of motor programming. Journal of Motor Behavior, 17(2), 190–218. 10.1080/00222895.1985.10735344 15140691

